# Gender-Specific Coagulation Profiles of Peripheral and Portal Blood May Help to Differentiate Malignant from Benign Pancreatic Tumour—Pilot Study

**DOI:** 10.3390/jcm11061573

**Published:** 2022-03-13

**Authors:** Aneta Szmiel, Alicja Majos, Wojciech Ciesielski, Anna Kumor, Janusz Strzelczyk, Krzysztof Szwedziak, Piotr Hogendorf, Adam Durczyński

**Affiliations:** 1 Department of General and Transplant Surgery, Medical University of Lodz, 4 Kosciuszki Street, 90-419 Lodz, Poland; szmielaneta@wp.pl (A.S.); alicja.majos@umed.lodz.pl (A.M.); wojciech.ciesielski@icloud.com (W.C.); janusz.strzelczyk@umed.lodz.pl (J.S.); krzysztof.szwedziak@umed.lodz.pl (K.S.); adam.durczynski@umed.lodz.pl (A.D.); 2 Department of Diagnostic Laboratory, Medical University of Lodz, 22 Kopcinskiego Street, 90-153 Lodz, Poland; anna.kumor@umed.lodz.pl

**Keywords:** pancreatic cancer, coagulation profile, portal blood, fibrinogen, aptt

## Abstract

Objective: Pancreatic adenocarcinoma (PDAC) and mass forming chronic pancreatitis (CP) can be easily misdiagnosed due to their resemblances in clinical, radiological, and biochemical criteria. In our previous study, we reported a very high concentration of D-Dimers in portal blood in patients with pancreatic cancer which may help to differentiate malignant from benign pancreatic tumours. In this study, we aim to describe other portal and peripheral coagulation profiles of PDAC in comparison to CP patients, as well to test the hypothesis; thus, it is possible to distinguish pancreatic malignancy and benign tumour based on these parameters. Methods: We included retrospectively 115 patients with the absence of venous thromboembolism (VTE), qualified to surgical treatment due to pancreatic tumours, both PDAC and CP. Patients underwent surgery in General and Transplant Surgery Unit of Medical University of Lodz between December 2011 and February 2014. Patients with distant metastases diagnosed before or during the surgery were excluded. The coagulation profile, which includes fibrinogen, activated partial thromboplastin time (aPTT), prothrombin time (PT), and thrombin time (TT), was determined in blood samples from the portal and peripheral vein taken intraoperatively. Results: The fibrinogen level was higher and the aPTT index shortened in the peripheral and portal blood of the PDAC group, which reflects the well-known link between PDAC and general hypercoagulability. Furthermore, these effects are sex-specific. The mean age in the CP group was lower than in the PDAC group (54.63 ± 12.37 vs. 63.77 ± 3.23, *p* < 0.001) and correlated with the fibrinogen distribution in male patients with CP (portal r = 0.34; *p* = 0.07; peripheral r = 0.39; *p* = 0.04). We calculated sex-specific logistic regression models (male: peripheral aPTT and age, AUC: 0.795, female: portal fibrinogen and age, AUC: 0.805), both maintaining the good discrimination properties after V-fold cross validation (0.759, 0.742). Conclusions: Our study shows that the differences between coagulation profiles in PDAC and CP patients not only seems to be a reflection of gender-specific biological features, but also helps to discriminate between them. The main goal of the study was to explore the biology of pancreatic cancer and lay a solid base for further investigations of PDAC biomarkers. This paper is the first to describe the detailed coagulation profile in portal blood in patients with pancreatic solid tumors. At present, the clinical application of our results is not clear; however, we hope that it may improve our understanding of this complex disease.

## 1. Introduction

Pancreatic cancer (PC) remains one of deadliest malignancies in US causing estimated 45,750 deaths compared with 56,770 new cases in 2019. Continuously, increasing incidence rate is still associated with diagnosis at a very late stage and very low 5-year survival rate (5–9%) [1]. Due to deficiency in early detection tools, even optimal treatment has a non-satisfying impact on survival. Unfortunately, prognosis for the next 40 years shows a linear increase in PC deaths, reaching almost 730,000 in 2060 [2]. There is still a lack of effective and cost-efficient screening tools. No single biomarker is sensitive and specific enough for early PC detection [3].

An important risk factor for PC is chronic pancreatitis (CP) [4]. Pancreatic adenocarcinoma (PDAC) and mass forming chronic pancreatitis (MFCP) can be easily misdiagnosed due to resemblances in clinical, radiological, and biochemical criteria. Imaging methods have variable sensitivity and specificity in differentiation of these two pathologies. Endoscopic ultrasound-guided fine-needle biopsy is the most dependable method; however, it is still invasive and not 100% sensitive [5]. Therefore, 5–15% of focal masses in the head of the pancreas preoperatively presumed to be malignant are classified as benign in post-surgery histopathological examination, which questions the validity of radical resection. Current clinical tools are not precise enough to differentiate PDAC from MFCP [6] and it facilitates research programs to search for novel PC markers.

Recent experimental and clinical studies have evidenced an association between cancer and blood haemostasis. D-dimer levels were reported to be significantly higher in patients with cancer and associated with clinical stages and metastases. We have reported a very high concentration of D-dimer in the portal blood of patients with pancreatic cancer which may help to differentiate malignant from benign pancreatic tumors [7]. Nevertheless, even the basic information about human portal blood coagulation times and fibrinogen concentration is lacking, not only the specific information about CP and PDAC patients characteristics in this field. With knowledge of these sex-specific differences which are widely investigated previously in the context of cardiovascular diseases (CVD), it is logical to assume similar differences between male and female portal coagulation profiles resulting from hormonal balance [8,9,10].

Thus, in this study, we aim to describe both portal and peripheral coagulation profiles of PDAC in comparison to CP patients, as well to test the hypothesis; thus, it is possible to distinguish pancreatic malignancy and benign tumors based on these parameters.

## 2. Materials and Methods

We retrospectively included 115 patients with the absence of venous thromboembolism (VTE) who qualified for surgical treatment due to pancreatic tumors, both PDAC and CP. Patients underwent surgery in General and Transplant Surgery Unit of Medical University of Lodz between December 2011 and February 2014. Patients with distant metastases diagnosed before or during the surgery were excluded.

Patients were qualified for surgical treatment due to presence of pancreatic tumors in imaging tests (CT, MRI, EUS, and USG), which was in line with the current treatment guidelines. All qualified for the operation patients were within normal nutritional status, preoperatively confirmed by adequate assessments.

The coagulation profile, which includes fibrinogen, activated partial thromboplastin time (aPTT), prothrombin time (PT), and thrombin time (TT), was determined in the blood samples intraoperatively taken from portal and peripheral veins.

Statistical analysis was conducted using Statistica 13 PL. Statistical significance was assessed at the 0.05 alpha level. We used the Student’s *t*-test or the Mann–Whitney test for two group comparisons, dependent from their normal distribution status and the Pearson coefficient for correlations. We also calculated logistic regression models, assessed using ROC curves and the V-fold cross validation method.

Our study was conducted in accordance with ethical standards of Helsinki Declaration. Approval was obtained from the Ethical Committee of the District Medical Chamber No RNN/32/19/KE.

## 3. Results

**Figure 1:**
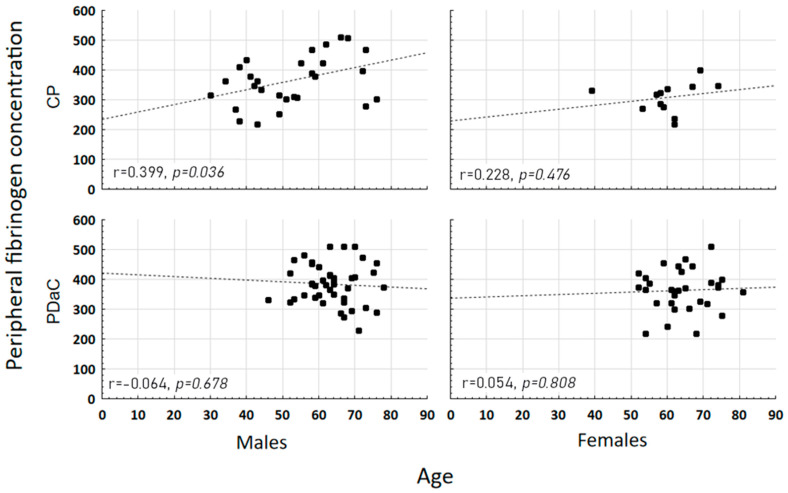
The correlations between peripheral fibrinogen concentration and age according to sex (male or female) and diagnosis (CP—chronic pancreatitis, PDaC—pancreatic adenocarcinoma). A positive correlation in males diagnosed with CP suggests a cause-and-effect relationship between the examined parameters in this group of patients.

**Figure 2:**
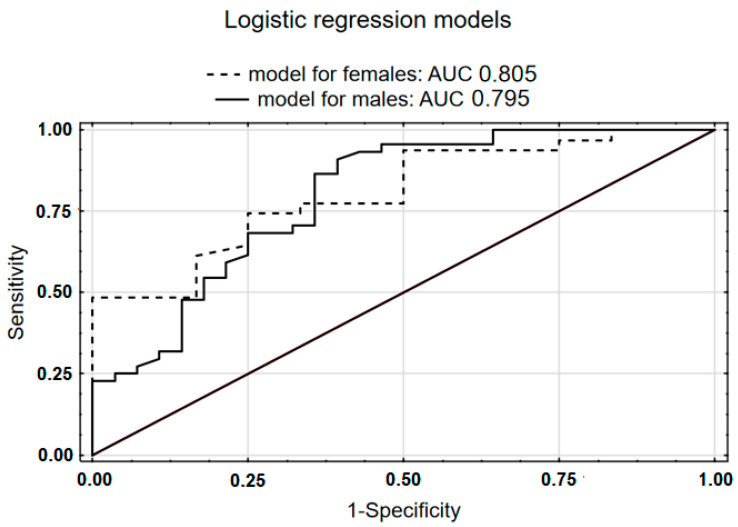
Sensitivity vs. 1-specificity chart for sex-specific logistic regression models. The models were based on the following parameters: peripheral aPTT and age for males, and portal fibrinogen and age for females. The course of curves as well as the AUC levels seem to be similar.

Histopathological examination of postoperative specimens revealed 75 PDAC (65.22%) and 40 CP (34.78%). Detailed basic statistics are presented in Table 1. All excised tumors were confined to the pancreas without infiltration of celiac trunk or superior mesenteric artery with average diameter of 3 cm. The CP group had mass forming chronic pancreatitis resulting from well-known multifactorial etiology. The whole group consisted of 72 males (62.61%) and 43 females (37.39%), both in PDAC and CP subgroups, though predominantly male (respectively: *n* = 44, 58.67%; *n* = 28, 70.00%, *p* = 0.232). Male and female PDAC patients did not statistically significantly differ in terms of grade (males: G1: 9.09%; G2: 90.91%; G3: 0.00%; females: G1: 5.26%; G2: 84.21%; G3: 10.53%; *p* = 0.276). Peripheral fibrinogen levels were higher and the aPTT index shortened in peripheral and portal blood of PDAC in comparison to CP patients (medians, respectively: peripheral fibrinogen: 374.00 vs. 336.00; *p* = 0.29; peripheral aPTT 30.50 vs. 32.05, *p* = 0.026; portal aPTT: 31.10 vs. 33.85, *p* = 0.009). Furthermore, we proved that these effects are sex-specific. Both portal and peripheral fibrinogen concentrations showed good differentiating value in females (medians; portal: PDAC: 339.00 vs. PC 270.50, *p* = 0.006; peripheral: PDAC: 366.00 vs. PC: 320.50, *p* = 0.01), while it was aPTT in males (medians; portal: PDAC: 31.70 vs. PC: 34.20, *p* = 0.042; peripheral: PDAC: 30.50 vs. PC: 32.50, *p* = 0.022). The details of these comparisons are presented in Table 2. The mean age in the CP group was also lower than in the PDAC group (54.63 ± 12.37 vs. 63.77 ± 3.23, *p* < 0.001), which correlated with the fibrinogen distribution in male patients with CP (portal r = 0.34; *p* = 0.07; peripheral r = 0.39; *p* = 0.04) (Figure 1). We calculated sex-specific logistic regression models (male: peripheral aPTT and age, AUC: 0.795, female: portal fibrinogen and age, AUC: 0.805) (Figure 2), both maintaining the good discrimination properties after V-fold cross validation (respectively: 0.759; 0.742).

## 4. Discussion

To discuss the possible reasons for the discrepancies between males, females, CP patients, and PDAC patients, it is important not to overlook any of the main coagulation control mechanisms involved. Obviously, there are well-known links between the presentation of a high peripheral fibrinogen concentration and female sex, as well as between hypercoagulability and cancer. The first results from the physiological actions of sex hormones. Testosterone has an inverse correlation with plasma fibrinogen, body mass index (BMI), and visceral fat. Both CP and PDAC patients are expected to have a slim build as a result of pancreatic disease; thus, a bigger weight loss should be linked to higher testosterone levels and a lower fibrinogen concentration. Women, who initially show higher fibrinogen concentration levels than men (median of median 3.6 in non-CVD, non-smoking women vs. 3.1 in non-CVD, non-smoking men, according to Ganotakis et al.) [9], are less prone to become heavy smokers in Poland [11]. This is also common in the CP male patients group [12], and could represent an adverse effect (non-CVD, smokers, men: median fibrinogen 3.8, according to Ganotakis et al.), as previously mentioned [9]. This fact can possibly be due to a lower = prognostic value of portal fibrinogen in the group of female patients.

Directly influencing the coagulation profile, especially in the portal blood, is fairly common; however, potentially disturbing the interpretation of this study results in tissue factor (TF) overexpression on the tumor cells [13]. One of its effects is well described by our D-dimer group with a high concentration in portal blood [7,14,15,16,17,18]; however, the other can shorten the aPTT and raise the level of fibrinogen concentration (Figure 2). Our study proved that another haemostatic marker for hypercoagulability, i.e., shortened aPTT, may also help to distinguish between different types of pancreatic tumors in males. aPTT is a screening test used when there is a deficiency of factors II, V, VIII, IX, X, XI, and XII in the intrinsic and common pathways [19]. However, there is a limited number of studies about the gender differences regarding aPTT levels, PT levels, and international normalized ratio (INR); thus, awareness of this remains mostly unknown. Therefore, the influence of sex on aPTT values and its utility in the differentiation of distinct pancreatic tumors require further analyses.

Up to now, a strong correlation between fibrinogen and enlarged tumor size, increased tumor growth, increased metastatic potential, and poor prognosis in various malignancies has been recognized [20]. Furthermore, oncologic patients have a higher risk of cancer progression and mortality when compared with patients with normal fibrinogen levels [21]. Additionally, preoperative plasma fibrinogen levels are an independent risk factor of subsequent tumor recurrence and overall survival [20]. Previous studies have proved that hyperfibrinogenemia is related with adverse pathological features and lymphatic invasion [22]. Another possible underlying mechanism is the direct activation of the clotting response, the production of procoagulant factors, and the indirect stimulation of mononuclear cells to secrete these factors [22]. Regardless of the exact mechanism, the authors agree on the prothrombotic potential of neoplastic diseases. The reasons for the discrepancy between the parameters of peripheral and portal blood seem to be more enigmatic.

There is not much knowledge about substance concentrations in portal blood. However, portal veins can collect blood directly from the pancreas to avoid the possible effect of tumor markers becoming inactivated in the liver. Thus, derived from the same concept and safety profile as islet cell transplantation by interventional radiology using Doppler imaging guidance, in which the portal vein is accessed percutaneously, emerging reports have centered on sampling and investigating portal blood in patients with PDAC. Recently, the utility of EUS-guided vascular access of the portal vein for diagnostic and therapeutic interventions has been evaluated with a satisfactory safety profile [23].

In humans, D-dimer portal concentration is proved to be higher than peripheral when comparing PDAC patients to those with benign pancreatic diseases [7,15,16,17,18]. Furthermore, in the pilot study on a small group of PDAC patients, the fT3 change coefficient (peripheral to portal blood) showed a strong statistically significant negative correlation with the portal D-dimer concentration level [14], which is in line with Durczynski et al. These results allude to the existence of multiple, multidirectional, mutual bindings between cancer culture, hormonal balance, and coagulation profile. Although the influence of neoplastic disease on the body’s hormonal balance is widely recognized, it is also highly knotty. Amongst others, the actions of leptin and adiponectin (also of sex-specific concentration) affect the level of fatty tissue in the organism, which not only indirectly influences previously discussed sex hormones level, but also its use as a biomarker of PDAC [24].

Thus far, researches mainly focused on d-dimers and fibrinogen concentration in peripheral blood. If combined, their low preoperative concentration (low-low group with fibrinogen ≤ 3.31 g/L, D-dimer ≤ 0.53 mg/L) was proven to have a predictive role in the overall survival in PDAC patients undergoing R0 radical pancreatoduodenectomy or distal pancreatectomy with splenectomy (any high group vs. low–low group, HR: 2.397, 95%CI: 1.723–3.335, *p* < 0.001). Authors found no statistical correlation between peripheral fibrinogen concentration and sex or age [25]. Zhan et al. investigated also preoperative fibrinogen-to-albumin ratio (FAR) in group of R0 resection PDAC patients. They reported FAR to be useful for improving preoperative prognosis in patients undergoing radical resection (HR: 2.257, 95%CI: 1.725–2.952, *p* < 0.001) [26]. Significantly elevated concentration of peripheral blood fibrinogen collected in the day of surgery in PDAC patients was investigated by Chung et al. (PDAC patients 3.08 ± 0.565 vs. 2.54 ± 0.249 log10 ng/mL in control group, *p* < 0.001). Authors described longer OS in patients with low fibrinogen concentration 489 days (95% confidence interval, 248.1–729.9) vs. 172 days (95% CI, 58.4–285.6) (*p* = 0.008)) [27]. To improve the diagnostic accuracy between PDAC and IPMN, Mattila et al. recommend combining the coagulation profile of peripheral blood (fibrinogen and factor VIII), alkaline phosphatase, and albumin with CA19-9. Such a panel of biomarkers improves the AUC of the ROC curve to 0.95 (95% CI: 0.89–0.99), in comparison with 0.80 (0.71–0.88) for CA 19–9 alone (*p* = 0.002) [28].

Although the overproduction of fibrinogen in PDAC has been documented [29], the potential clinical utility of hyperfibrinogenemia to distinguish PDAC from CP remains unknown. The current study is the first to discover that high portal and peripheral fibrinogen levels may help to differentiate malignant from benign pancreatic tumors, particularly among females. This may result in the introduction of new diagnostic tools, for example, portal blood sampling by means of endoscopic ultrasound, ultrasound, or laparoscopic. However, it is more invasive, which could help to avoid an extensive and unnecessary surgical treatment. This study also supports the relation between hyperfibrinogenemia and PC, although the mechanisms of fibrinogen overproduction may differ in males and females. Recent studies in cancer biology indicate that sex differences may have a significant impact on cancer cells and systems. These discrepancies in gender-specific cancer biology may even affect how males and females respond to therapy [30]; therefore, further studies are needed in this area.

We are aware of some limitations of our study. First, the tested group is relatively large enough to demonstrate the statistical importance of the results, but imbalanced between PDAC and CP subgroups. Second, due to the retrospective character of this study, some information on patients’ characteristics was incomplete. Because of this, the data on the location, size, grade, and stage of PDAC are limited. The coagulation profile is affected by many factors; thus, more precise details regarding patient-specific clinical parameters are needed (e.g., BMI index, hormone levels, history of Tabaco and alcohol consumption, albumin level, total blood count). Moreover, the described differences in portal and peripheral blood parameters, although statistically significant, cannot be yet implied clinically. Further studies are required.

## 5. Conclusions

To conclude, our study shows that the differences between coagulation profiles in PDAC and CP patients not only seems to be a reflection of gender-specific biological features, but also helps to discriminate between them. In addition, this paper is the first to describe the detailed coagulation profile in portal blood in patients with pancreatic solid tumors. The main goal of the study was to explore the biology of pancreatic cancer and lay a solid base for further investigations of PDAC biomarkers. We believe that further analysis of portal blood and coagulation parameters as diagnostic tools in PDAC and diagnostic tools of PDAC is an interesting path to follow. At present, the clinical application of our results is not clear; however, we hope that it may improve our understanding of this complex disease. 

## Figures and Tables

**Table 1:** Basic statistics of tested parameters values in PDAC and CP groups.

Parameter	PDAC				CP				*p*
	*n*%				*n*%				
Sex									0.232
Male	44 (61.11%)				28 (38.89%)				
Female	31 (72.09%)				12 (27.91%)				
	Mean ± SD	Median	Min.	Max.	Mean ± SD	Median	Min.	Max.	
portal blood									
Fibrinogen [mg/dL]	336.44 ± 90.00	339.00	141.00	624.00	302.18 ± 105.72	293.50	85.00	592.00	0.046 †
PT [s]	16.97 ± 3.08	16.50	12.70	26.50	18.27 ± 6.86	16.70	12.10	49.60	0.923
PR	80.96 ± 13.24	81.00	54.50	107.00	80.68 ± 14.31	80.50	54.50	110.30	0.918
INR	1.33 ± 0.27	1.28	0.92	1.86	1.34 ± 0.29	1.32	0.86	1.86	0.881
TT [s]	15.85 ± 2.40	15.50	10.90	20.80	16.17 ± 2.30	16.25	11.10	20.80	0.307
aPTT [s]	31.59 ± 4.26	31.10	23.00	41.30	34.08 ± 4.84	33.85	23.00	41.30	0.009 *
aPTT-R	0.96 ± 0.13	0.96	0.69	1.26	1.04 ± 0.15	1.04	0.69	1.26	0.006 *
TT-R	1.17 ± 0.18	1.16	0.83	1.54	1.20 ± 0.16	1.16	0.84	1.54	0.257
peripheral blood									
Fibrinogen [mg/dL]	375.54 ± 68.25	374.00	220.00	511.00	347.35 ± 78.13	336.00	220.00	511.00	0.029 *
PT [s]	14.55 ± 1.14	14.30	12.30	16.50	14.36 ± 1.06	14.20	12.30	16.50	0.470
PR	91.80 ± 7.62	92.80	77.30	107.00	93.37 ± 7.30	94.40	77.30	107.00	0.251
INR	1.11 ± 0.11	1.09	0.90	1.32	1.09 ± 0.10	1.08	0.90	1.32	0.437
TT [s]	14.16 ± 1.70	13.90	11.30	18.20	14.41 ± 1.93	14.00	10.70	18.20	0.555
aPTT [s]	30.44 ± 3.56	30.50	23.50	38.20	32.13 ± 3.70	32.05	23.50	38.20	0.026 *
aPTT-R	0.92 ± 0.10	0.93	0.73	1.15	0.98 ± 0.11	0.98	0.73	1.15	0.018 *
TT-R	1.04 ± 0.13	1.04	0.84	1.37	1.07 ± 0.14	1.04	0.86	1.37	0.351
Age [y]	63.77 ± 3.23	63.00	46.00	81.00	54.63 ± 12.37	57.50	30.00	76.00	≤0.001 *

†—*p* < 0.1, *—*p* < 0.05. PDAC—pancreatic adenocarcinoma, CP—chronic pancreatitis, PT—prothrombin time, PT—prothrombin time, INR—international normalized ratio (the ratio of a patient’s PT to a normal sample, raised to the power of the ISI value for the analytical system being used), TT—thrombin time, aPTT—activated partial thromboplastin time, aPTT-R—aPTT ratio (the ratio of patient’s aPTT to the mean of laboratory control aPTT), TT-R—TT ratio (the ratio of the patient’s clotting time to the mean of the laboratory control clotting time), min.—minimum, max.—maximum, bold font—statistical significance. PR—the ratio of a patient’s measured PT to the normal laboratory reference PT.

**Table 2:** Tested parameters in CP and PDAC groups, according to the patient’s sex. *p* level refers to U tests.

Parameter	CP				PDAC				*p*
	Mean ± SD	Median	Min.	Max.	Mean ± SD	Median	Min.	Max.	
Men									
Portal fibrinogen [mg/dL]	316.29 ± 120.25	302.00	85.00	592.00	328.43 ± 82.21	328.50	152.00	544.00	0.583
Peripheral fibrinogen [mg/dL]	364.25 ± 82.24	363.50	220.00	511.00	383.98 ± 66.88	382.50	230.00	511.00	0.260
Portal aPTT [s]	34.62 ± 5.11	34.20	23.00	41.30	32.05 ± 4.37	31.70	23.30	41.30	0.042
Peripheral aPTT [s]	32.89 ± 3.66	32.50	25.10	38.20	30.69 ± 3.61	30.50	23.90	38.20	0.022
Age [y]	52.39 ± 13.13	52.00	30.00	76.00	63.50 ± 7.15	63.00	46.00	78.00	≤0.001
Women									
Portal fibrinogen [mg/dL]	269.25 ± 49.44	270.50	189.00	350.00	347.80 ± 100.33	339.00	141.00	624.00	0.006
Peripheral fibrinogen [mg/dL]	307.92 ± 51.15	320.50	220.00	400.00	363.55 ± 69.47	366.00	220.00	511.00	0.010
Portal aPTT [s]	32.82 ± 4.08	33.30	24.60	39.50	30.94 ± 4.07	30.80	23.00	39.00	0.193
Peripheral aPTT [s]	30.35 ± 3.27	30.30	23.50	34.90	30.08 ± 3.50	30.70	23.50	38.20	0.695
Age [y]	59.83 ± 8.75	59.50	39.00	74.00	64.14 ± 7.65	63.00	52.00	81.00	0.159

PDAC—pancreatic adenocarcinoma, CP—chronic pancreatitis, aPTT—activated partial thromboplastin time, min.—minimum, max.—maximum, underline—*p* < 0.05.
